# Evaluation of Nanocomposite Made of Polylactic Acid and Nanocellulose from Carrot Pomace Modified with Silver Nanoparticles

**DOI:** 10.3390/polym12040812

**Published:** 2020-04-04

**Authors:** Monika Szymańska-Chargot, Monika Chylińska, Piotr M. Pieczywek, Anna Walkiewicz, Giorgia Pertile, Magdalena Frąc, Krystian J. Cieślak, Artur Zdunek

**Affiliations:** 1 Institute of Agrophysics PAS, Doswiadczalna 4, 20-290 Lublin, Poland; m.chylinska@ipan.lublin.pl (M.C.); p.pieczywek@ipan.lublin.pl (P.M.P.); a.walkiewicz@ipan.lublin.pl (A.W.); g.pertile@ipan.lublin.pl (G.P.); m.frac@ipan.lublin.pl (M.F.); a.zdunek@ipan.lublin.pl (A.Z.); 2 Faculty of Environmental Engineering, Institute of Renewable Energy Engineering, Lublin University of Technology, Nadbystrzycka 40B, 20-618 Lublin, Poland; k.cieslak@pollub.pl

**Keywords:** polylactic acid, nanocellulose, silver nanoparticles, composites, mechanical properties, antibacterial properties

## Abstract

In this research, it was proposed to use carrot cellulose nanofibrils (CCNF) isolated from carrot pomace modified with silver nanoparticles (AgNPs) as a filler of polylactic acid (PLA) composites matrix. The new procedure was based on two steps: first, the preparation of nanocellulose modified with metal nanoparticles, and then the combination with PLA. Two concentrations—0.25 mM and 2 mM—of AgNO_3_ were used to modify CCNF. Then, PLA was mixed with the filler (CCNF/AgNPs) in two proportions 99:1 and 96:4. The influence of CCNF/AgNPs on mechanical, hydrophilic, thermal, and antibacterial properties of obtained nanocomposites was evaluated. The greatest improvement of mechanical properties was observed for composite containing CCNF with 2 mM of AgNPs, which obtained the lowest Young modulus and highest strain at break. The degradation temperature was lower for PLA with CCNF/AgNPs, but crystallization temperature wasn’t influenced. The addition of CCNF/AgNPs also increased hydrophilicity. The transmission rates of oxygen, nitrogen, and carbon dioxide also increased after the addition of CCNF/AgNPs to PLA. The antibacterial function against *Escherichia coli* and *Bacillus cereus* was obtained after the addition of AgNPs but only at the contact surface with the material made, suggesting the lack of migration of nanoparticles from the composite.

## 1. Introduction

The main function of food packaging is to maintain the quality and safety of food products during storage, transportation, and to extend the shelf-life of food products by preventing unfavorable factors or conditions, such as spoilage microorganisms, chemical contaminants, oxygen, moisture, light, or external force. In order to perform such functions, packaging materials provide physical protection and create proper physicochemical conditions for products [1]. Nowadays, people are facing fast climate change and the problem of wastes utilization, connected with that. Therefore, reducing petroleum non-biodegradable polymers, such as polyethylene (PE), low- (LDPE) and high-density polyethylene (HDPE), or polypropylene (PP), usually used for producing food packaging, is an urgent need [2]. The ideal packaging materials are biodegradable and obtained from renewable biological resources, usually called biopolymers, with proper mechanical and barrier properties. Biopolymers have been considered as a potential environmentally-friendly substitute for the use of non-biodegradable and non-renewable plastic packaging materials. Thus, the efforts to develop new composites based on biodegradable polymers, for instance, polylactic acid (PLA), polyhydroxybutyrate (PHB), and other aliphatic polyesters, which are synthesized from renewable bio-based monomers, have increased [3]. Among all these polymers, the most commonly used are polylactic acid composites. PLA is widely produced by bacterial fermentation of sugar beet or corn starch and has acceptable properties like transparency or stiffness and is easily printed [3,4]. On the other hand, some properties, such as flexibility, toughness, permeability, or thermal properties of PLA, still need to be improved [5]. One of the methods of adjusting PLA properties to a specific application is introducing some reinforcing agents, such as clays, silicates, carbon nanotubes, cellulose/nanocellulose, or chitin/chitosan [6]. Especially, nanocellulose due to its unique properties, high availability, originating from renewable plant-based sources, biocompatibility, and biodegradability is widely used as reinforcement for packaging materials. Nanocellulose is cellulose microfibrils, disintegrated by mechanical or acid treatment, and usually occurs in thin and long nanofibrils (CNF) or short nanocrystals (CNC) forms [7]. The presence of nanocellulose in the PLA matrix enhances its crystallization and also speeds up the process of composite disintegration [4]. As a recent example, it has been shown that multifunctional bionanocomposite films based on PLA-PHB reinforced with CNC or surfactant-modified CNCs show better degradability without a loss in their transparency [8]. While the addition of metal nanoparticles to composites gives them antibacterial properties, which can be used as an active packaging [9]. So far, most of the efforts have been focused on the preparation of nanocomposite films based on the PLA matrix with the reinforcement of cellulose modified with surfactant and silver nanoparticles treated as an active additive with biocide activity. The substantial reduction in water vapor permeability and oxygen transmission rate (OTR) has been observed [10]. In addition, the ternary nanocomposite (PLA/CNC/Ag) has shown better mechanical properties and a faster degradability rate than a binary one (PLA/CNC) [11,12].

However, some obstacles are needed to be overcome. One of them is connected with the hydrophilicity of nanocellulose, which has an influence on compatibility with hydrophobic polymers and can result in weaker matrix-filler interaction [6]. The other problem is the migration rate of nanoparticles. Nanoparticles are now becoming widely used in many fields of industry, including food packaging. Metal nanoparticles recently have been considered a negative factor since substances are likely to constitute a danger to human health and/or alter the composition of foodstuff in an unacceptable manner. The European Food Safety Authority (EFSA) has evaluated the use of several silver-based substances intended to have contact with foods and defined a general specific migration limit of 0.05 mg of silver per kg of food [13]. However, some studies have shown that either inside the polymer or in aqueous solutions, the amount of silver necessary to affect the bacterial growth is in the range of 0.001–0.01 mg of Ag per kg of food [10]. Therefore, to see the biotic effects of AgNPs, we could use quantities lower than limits imposed by EFSA.

So far, the existing methods of ternary nanocomposites are a simple blend of biopolymer, nanocellulose, and metal/metal oxide nanoparticles. In the present study, nanocellulose modified with silver nanoparticles as the filler of the PLA matrix was used. The proposed approach involved first the preparation of nanocellulose modified with metal nanoparticles and then the combination with the biopolymer. Recently, the feasibility of using fruit and vegetable pomaces as the source of cellulose and nanocellulose has been demonstrated [14,15]. It has also been shown that the pomace source can lead to the acquisition of nanocellulose with different structures, i.e., high-intensity ultrasonication results in the formation of nanofibrils from apple pomace, while large whiskers are obtained from carrot pomace [15]. In the present study, the nanocellulose produced by a high-intensity ultrasonication process from carrot pomace that had an average diameter of 3.31 nm and a crystallinity degree of 80.09% was used [15]. The silver nanoparticles were directly synthesized and stabilized by nanocellulose present in the dispersion. There are different methods of preparing composites: electrospinning, solvent casting, or thermal forming from extruded pellets [4,8,10,11,16,17]. The final nanocomposite of PLA/nanocellulose/silver nanoparticles was obtained by solvent casting. The main goal of this experiment was to demonstrate the influence of different concentrations of nanocellulose and silver nanoparticles on the mechanical, antibacterial, thermal, and structural properties of the nanocomposites.

## 2. Materials and Methods

### 2.1. Materials

All chemicals: silver nitrate (AgNO_3_), sodium borohydride (NaBH_4_), hydrochloric acid (HCl), sodium hydroxide (NaOH), sodium hypochlorite (NaClO), dichloromethane (DCM) were purchased in POCH Poland (Avantor Performance Materials Poland S.A., Gliwice, Poland) and were of analytical grade. Polylactic acid (PLA) in the form of 3 mm granules was purchased in GoodFellow (Cambridge, UK). Deionized water with a conductivity of 0.11 µS/cm was used in all processes.

Nanocellulose was prepared from carrot pomace. The procedure is described in detail in our previous papers [15,18]. Briefly, the thermochemical process was based on the extraction of pectic and hemicellulosic polysaccharides in first acidic and then alkaline environments followed by lignin removal in sodium hypochlorite solution. After the treatment, carrot cellulose was obtained as water suspension with concentration around 4 wt.% The nanocellulose from cellulose was prepared via an ultrasonication treatment with a Sonics Vibracell ultrasonic homogenizer (VCX-130FSJ; Sonics & Materials, Inc., Newtown, CT, USA). The sonication system contained a temperature probe, and to avoid heating the samples, an ice bath was used. The operation amplitude of ultrasonic homogenizer was maintained at 90% of the nominal amplitude [15]. Finally, carrot cellulose nanofibrils (CCNF) at a concentration of 0.1 wt.% was obtained.

### 2.2. Silver Nanoparticles Synthesis

A sodium borohydride was added to the suspension of CCNF in water (0.1%) to obtain 2 mM NaBH_4_ concentration in the solution [19]. The initial temperature of CCNF was 20 °C. The solution was mixed using a magnetic stirrer for 20 min until the complete dissolution of NaBH_4_. Thereafter, 3 mL of an AgNO_3_ solution in chosen concentration (0.25 mM or 2 mM) was added dropwise to the CCNF/NaBH_4_ suspensions. The reaction was continued for 48 h at 20 °C with simultaneous mixing and no light. Gas bubbles visible during the first hours of synthesis originated from the hydrogen as a result of the reaction between sodium borohydride and water.

### 2.3. Preparation of Composites

Dichloromethane (DCM) was chosen as it is a good PLA solvent. As the CCNF/AgNPs dispersions were prepared in water, the exchange of solvent from water to DCM to obtain the PLA/nanocellulose composites was necessary. The final dispersion of CCNF/AgNPs in DCM was uniform without a trace of precipitation of nanocellulose. First, the water suspension of CCNF, CCNF/0.25AgNPs, and CCNF/2AgNPs was thickened by vacuum filtration (pump Basic 36, AgaLabor, Warsaw, Poland). A 0.65-μm-pore-diameter PDF membrane filter (EMD Millipore™ Durapore™; ϕ = 90 mm, Merck KGaA, Darmstadt, Germany) was used. Then, the obtained gel was suspended in acetone, mixed, and afterward centrifuged (6000 rpm, SIGMA 3-BOKS, Osterode am Harz, Germany). The solvent was poured over the suspension, and once again acetone was added, and centrifugation was repeated. Then, DCM was added to the residue, and centrifugation was repeated. The process was repeated twice. The final suspensions of CCNF or each CCNF/AgNPs in DCM had a concentration of 1%. The prepared CCNF/AgNPs in DCM was mixed with a 10% solution of PLA in DCM in two weight proportions of PLA to CCNF: 99:1 or 96:4 (Table 1). The 7 g of final dispersion was put on the aluminum dish (ϕ = 90 mm) and left to dry at room temperature. The final weight of each composite film was 0.5 g with a thickness of 0.2 mm. The obtained composites are presented in Figure S1. The addition of CCNF made films more opaque, and CCNF/AgNPs gave the yellow color of films.

### 2.4. Characterization of CCNF/AgNPs Colloids

#### 2.4.1. Spectroscopy UV-Vis

Spectrophotometric studies on the formed silver nanoparticles (AgNPs) were carried out. The absorbance of CCNF/AgNPs colloids was characterized by Genesys 10s UV-Vis (Thermo Scientific, Waltham, MA, USA). Quartz cuvettes with the optical path of 10 mm were used. The absorbance spectra were recorded over the range of 190–1000 nm. The 0.1% CCNF suspension was used as a reference sample. The measurements were taken after 48 h from adding a solution of silver nitrate.

#### 2.4.2. Atomic Force Microscope (AFM)

The CCNF/AgNPs were suspended in ultrapure water (Millipore, Burlington, MA, USA) to obtain the 0.01 wt.% concentration. Then, the suspension was deposited onto a microscopic slide and dried in room condition at 23 °C for 24 h. The samples were kept in a desiccator before atomic force microscope (AFM) observations. Bioscope Catalyst II, supported with a Nanoscope V controller (Bruker, Billerica, MA, USA), was used for imaging in the semiautomatic tapping mode (ScanAsyst imaging mode). A silicon nitride cantilever (Bruker ScanAsyst AIR, Billerica, MA, USA) with a nominal radius of pyramidal tip 2 nm, spring constant of 0.4 N·m^−1^, and the resonance frequency of 70 kHz was used. The experiment was performed in ambient air at room temperature of about 20–22 °C and relative humidity (RH) of 26%–30%. Scan area 4 μm^2^ (aspect ratio 1:1) and image resolution 512 × 512 points were set. The scan rate of 0.5 Hz was maintained for obtaining appropriate image quality. For each sample, around 5 images from various regions were collected.

### 2.5. Composite Characterization

#### 2.5.1. Fourier Transform Infrared Spectroscopy (FTIR)

Fourier transform infrared spectroscopy (FTIR) spectra were collected in the Nicolet 6700 Fourier Transform infrared spectrometer (FTIR, ThermoScientific, Waltham, MA, USA). The Smart iTR ATR sampling accessory was used. The composite films were directly placed on the ATR crystal and measured. The spectra were collected over the range of 4000–650 cm^−1^. For each material, 3 samples under the same conditions were examined. For each sample, 200 scans were averaged with a spectral resolution of 4 cm^−1^. Next, for a given material, the final average spectrum was calculated. These spectra were normalized to 1 at 1081 cm^−1^ (C–O stretching vibration). All the spectra manipulation was carried out using The Origin Pro 8.5 (OriginLab Corporation, Northampton, MA, USA).

#### 2.5.2. Scanning Electron Microscopy (SEM)

The morphology of the composite surface was examined by scanning electron microscopy (SEM; Hitachi SU3500, Tokyo, Japan) at 5 kV under high-vacuum conditions. The small cut of each sample was applied to the aluminum stage covered by carbon tape. Next, the samples were coated with an ultrathin gold layer (Au) using an ion-sputtering machine (Cressington Sputter Coater 108 Auto, Watford, UK).

#### 2.5.3. Differential Scanning Calorimetry (DSC)

DSC analysis was performed using a TA Instruments DSC 250 system (Waters Corporation, Milford, MA, USA), and 5–10 mg of the film’s samples were sealed in aluminum pans, and a 20–400 °C heating increased under a nitrogen flux of 50 mL min^−1^; the heating rate was 10 °C min^−1^. The analyses were preceded by scans in the heat-cool-heat mode with a maximum temperature at 200 °C to eliminate water from the sample but not cause its decomposition. The data were analyzed using Trios v.4.2.1 (TA Instruments, Waters Corporation) software. The enthalpy was determined as the area under the peak of the curve in the range 240–380 °C.

#### 2.5.4. Wettability

The contact angles of 5 μL water droplets on the film’s surface attached to microscopic slides by double-sided tape (T: 23.5 ± 0.1 °C; RH: 31.6%) were measured. The Rame Hart 200Std goniometer (Rame Hart Instrument co., Succasunna, NJ, USA) was used. The measurements were performed for 10 min with 1-s time intervals. A longer measurement did not give any reliable results, and it was decided to be completed after 10 min. Each contact angle value corresponded to the mean value of the left and right contact angle at a given point in time.

#### 2.5.5. Gas Transmission Measurements

The N_2_, O_2_, and CO_2_ permeation measurements were conducted with a custom-designed permeation system using the constant-volume/variable-pressure method. A two-chamber vessel as separated by a film of tested composite (diameter 30 mm) was used as part of the measurement system. Priori each measurement, both chambers were refluxed by helium (He), and the excess gas was discharged using a second needle. Then, one chamber was filled with investigated gas, and the other was denoted as a reference chamber. The certified gas standard (0.5% CO_2_; 98.5% N_2_; 0.5% N_2_O; 0.5% CH_4_; AirLiquide, Krakow, Polska) was used to obtain elevated CO_2_ and N_2_ concentrations, while ambient air was added for O_2_ tests. At the same time, the excess of added gas was removed to balance the tension; therefore, the atmospheric pressure was in both chambers (monitored with an Infield 7 m, UMS, München, Germany). The gas from a reference chamber was collected with a syringe (injection 200 μL) every minute until the pressure in both chambers was the same and then dosed for quantitative and qualitative analysis. A gas chromatograph (Shimadzu GC-14A, Kyoto, Japan) equipped with a thermal conductivity detector (TCD) with a column (3.2 mm diameter) packed with Porapak Q (for CO_2_) and Molecular Sieve 5A (for O_2_ and N_2_) was used to determine the CO_2_, N_2_, and O_2_ concentration. The temperature of the detector and the column was 60 °C and 40 °C, respectively. Helium (40 cm^3^ min^−1^) was used as a carrier gas [20]. The measurement was conducted at a temperature range of 21–23 °C and RH 21%–22 % [21]. The average rate of gas transmission rate was calculated based on the changes in its concentration divided by the time. The measurements were performed at least in triplicate, and the mean value was presented.

#### 2.5.6. Mechanical Properties

All the composites under the study were tested for tensile strength, Young’s modulus, and elongation at break. Samples of composites were prepared as rectangular strips with a length of 40 mm and a width of ~2 mm. Precise measurements of the sample width were carried out using the Olympus SZX16 (Olympus Corporation, Tokyo, Japan) microscope with an SDF PLAPO 0.5 XPF lens, equipped with a DFK 51BU02.H digital camera (The Imaging Source Europe GmbH, Bremen, Germany). Image resolution was equal to 8.26 µm per pixel. The width of each sample was calculated as the mean value from three measurements. The thickness of each sample was measured using a digital micrometer BAKER IP54 (Baker Gauges India Private Limited, Pune, India) with a measurement accuracy equal to 0.001 mm. The cellulose strips were subjected to uniaxial tensile testing using a miniature tensile stage (Deben Microtest, Suffolk, UK). The initial gap between grips was equal to 10 mm. The mechanical experiments were carried out up to sample rupture with a deformation speed of 0.2 mm min^−1^. Tensile force and elongation of the sample were recorded and converted into stress and strain, respectively. The stress was determined as the ratio of the tensile force to the sample’s cross-sectional area. The strain was defined as the ratio of the sample elongation to its initial length. The Young’s modulus was determined as the slope of the longest linear part of the stress–strain curve. The mechanical tests were repeated ten times for each nanocellulose sample.

#### 2.5.7. Antimicrobial Assay

In order to analyze the microbial growth inhibition by nanocellulose and silver nanoparticles, three different species of bacteria (*Staphylococcus epidermidis, Escherichia coli, and Bacillus cereus*) were selected from microorganisms’ collection of the Laboratory of Molecular and Environmental Microbiology, Institute of Agrophysics, Polish Academy of Sciences (Lublin, Poland). The information about the microorganisms and the conditions of pre-culturing are presented in Table S1. All microorganisms were incubated and shaken at 180 rpm for 24 h at 37 °C. The antimicrobial properties were determined by measurements of the inhibition zone of bacteria using the modified Kirby–Bauer Disk diffusion susceptibility test protocol [18]. Each Petri dish was inoculated with 300 µL of final cell suspension of bacterial strains. The initial number of bacteria was approximately 107–108 CFU/mL (colony-forming unit per mL), with OD (optical density) of 1 measured at 610 nm. The OD was recorded by Infinite^®^ M200PRO spectrophotometer (Tecan, Männedorf, Switzerland). After the bacterial suspension was dry, we applied, in each quarter of the Petri dish, a disc of each analyzed nanocomposite (the dimension of each disc was 5 mm).

Inoculated Petri dishes were incubated at 37 °C. The diameter of the inhibition zone (in mm) was measured every 24 h for 7 days. The antibacterial activity was evaluated by measuring the diameter of the inhibition zone (mm) on the surface of plates. Each treatment was prepared in three replicates.

#### 2.5.8. Statistical Analysis

The significant differences were calculated by a posthoc analysis using the Tukey test. All the statistical analyses were performed using STATISTICA 10.0 software (StatSoft, INC., Tulsa, OK, USA).

## 3. Results and Discussion

### 3.1. Nanocellulose Modified with Silver Nanoparticles

**Figure 1:**
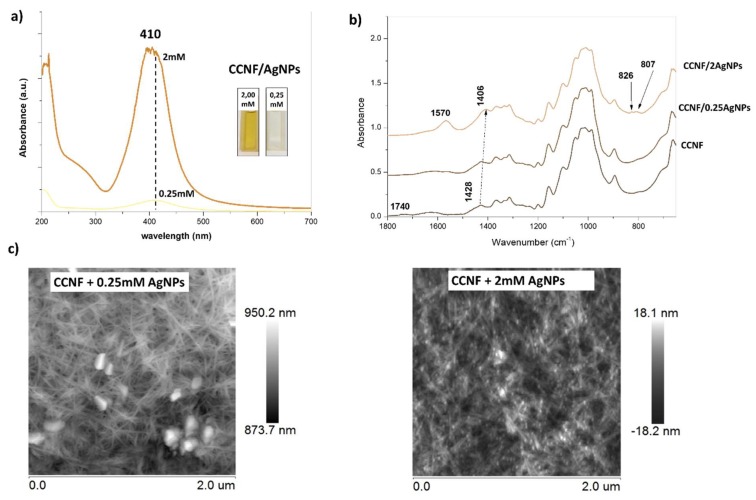
(**a**) UV-Vis spectra of CCNF/AgNPs 0.25 and 2 mM dispersions in water. (**b**) FTIR spectra of CCNF, CCNF/AgNPs 0.25 mM and 2 mM in the range 1800–650 cm^−1^. (**c**) AFM images of CCNF/AgNPs 0.25 and 2 mM with visible silver nanoparticles attached to nanocellulose.

The 0.1% nanocellulose modified with AgNPs in two concentrations, 0.25 mM and 2 mM, was prepared. Both colloids were yellowish in color. The UV-Vis spectra had one dominated maximum absorbance at 410 nm, which was evidence of the formation of spherical nanoparticles (Figure 1a) [22]. Moreover, FTIR spectra were recorded to check whether there was an interaction between synthesized nanoparticles and nanocellulose (Figure 1b). The biggest difference between CCNF and CCNF modified with AgNPs was the disappearance of band at 1740 cm^−1^ and the appearance of the band at 1570 cm^−1^, suggesting possible coordination bonding between carboxylic groups on nanocellulose surface and newly synthesized AgNPs [22]. Additionally, AFM images of CCNF/AgNPs were obtained with visible silver nanoparticles attached to the cellulose nanofibrils (Figure 1c). There was also evidence of synthesis of silver nanoparticles in different sizes: for 0.25 mM, the silver particles were significantly larger than those obtained for 2 mM.

### 3.2. Characterization of Composites

#### 3.2.1. FTIR

**Figure 2:**
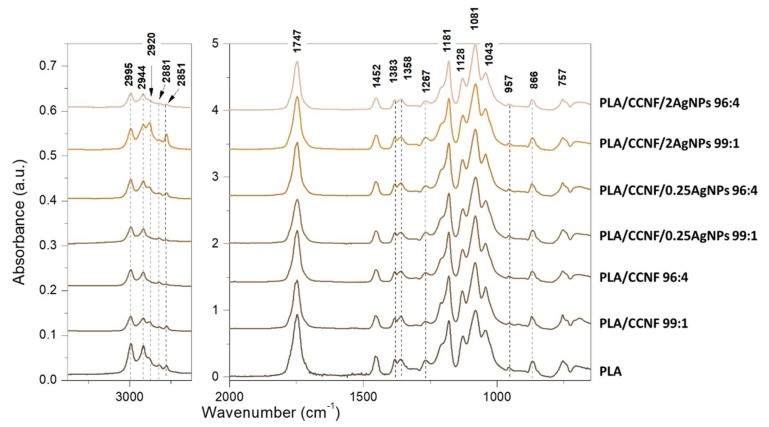
FTIR spectra of obtained nanocomposites of PLA and CCNF with or without AgNPs in concentration 0.25 mM and 2 mM presented in the range 3300–650 cm^−1^, with a break from 2750–2000cm^−1^.

FTIR spectra of PLA/CCNF/AgNPs composites are presented in Figure 2. This analysis attempted to characterize the incorporation of CCNF/AgNPs into the PLA matrix and distinguish the infrared bands and vibration shifts related to interactions between components. The absorption peaks in region 2995–2851 cm^−1^ of the PLA film spectrum are assigned mainly to the symmetric and asymmetric stretching vibration of the CH in –CH_3_ group [5]. A strong absorption band was observed at 1747 cm^−1^ due to the C=O stretching from the ester group in PLA, and the region between 1452–1358 cm^−1^ can be assigned to symmetric and asymmetric bending vibrations of –CH_3_ group [23]. The bands at 1267 and 1181 cm^−1^ are attributed to the symmetric and asymmetric stretching of C–O–C [24]. Bands at 1128, 1081, and 1043 cm^−1^ have been previously assigned to C–O stretching vibrations [25]. The bands at 868 and 757 cm^−1^ can be evidence of amorphous and crystalline phases of PLA, respectively [23]. The most striking feature was the lack of differences in spectra between pure PLA film and composites with CCNF or CCNF/AgNPs, which might be evidence of a lack of interactions between components.

#### 3.2.2. Morphology of Obtained Composite

**Figure 3:**
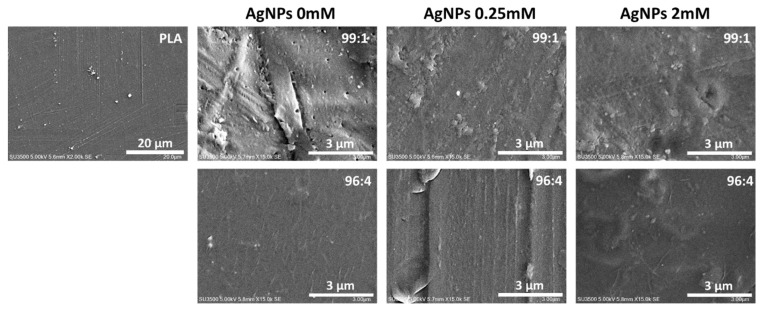
Scanning electron microscopy (SEM) micrographs of PLA (2000×) and composites with CCNF and CCNF/AgNPs (15,000×).

The morphology of the composite was evaluated by scanning electron microscopy (SEM) (Figure 3). The SEM micrograph of PLA film showed a very uniform, smooth, and homogeneous surface. After the addition of CCNF at a concentration of 1 % wt., the surface appeared to be rough with visible voids and agglomerates. The similar micrographs were obtained for samples PLA/CCNF/0.25AgNPs 99:1 and PLA/CCNF/2AgNPs 99:1. In both cases, the surface was rough with visible aggregates; however, the sample PLA/CCNF/2AgNPs 99:1 seemed to be more uniform compared with PLA/CCNF/0.25AgNPs 99:1. This suggested that smaller silver nanoparticles (Figure 1c) improved the dispersion of filler in the PLA matrix. In the case of samples with a higher amount of filler (96:4), micrographs presented a smoother surface with uniformly dispersed visible rod-like structures. For samples with CCNF/AgNPs (0.25 and 2 mM) apart of nanocellulose fibrils, some aggregates were also visible, but still, the surface of PLA/CCNF/0.25AgNPs 96:4 and PLA/CCNF/2AgNPs 96:4 was smoother than the 99:1 sample set.

#### 3.2.3. DSC

Differential scanning calorimetry (DSC) was used to study changes in the thermal properties of the PLA matrix after the addition of CCNF/AgNPs. DSC thermal properties, such as glass transition temperature (*T*_g_), melting temperature (*T*_m_) and enthalpy (*E*_m_), crystallization temperature (*T*_c_) and enthalpy (*E*_c_), as well as decomposition temperature (*T*_d_) and enthalpy (*E*_d_), are presented in Table 2. The glass transition temperature of PLA film was 57.8 °C, and the addition of CCNF or CCNF/AgNPs didn’t cause any change. These results were in good agreement with data obtained for ternary systems of PLA, cellulose nanocrystals, and silver nanoparticles [11,17]. The crystallization temperature of nanocomposites slightly increased by 1–2 °C compared with the neat PLA film. Previously, Cacciotti et al. (2014) presented that the decrease of the crystallization temperature of ternary nanocomposites might suggest a better dispersity of nanofiller [11]. On the other hand, the melting temperature of nanocomposite slightly decreased in comparison to the PLA film. The greatest change was the decrease in degradation temperature from 369 °C (neat PLA) to 348 °C after the addition of nanofiller to PLA matrix. Evidently, the amount of CCNF modified with silver nanoparticles had the greatest impact on decreased T_d_. Cacciotti et al. (2014) showed that nanocellulose combined with Ag promoted rearrangements of the PLA chains and formation of smaller crystals, which, in turn, led to lowering of melting temperature (by 1–2 °C) [11]. This process could have an effect also on degradation temperature. In this research, both melting and degradation temperatures decreased after the addition of CCNF modified with AgNPs.

#### 3.2.4. Hydrophobic Properties

**Figure 4:**
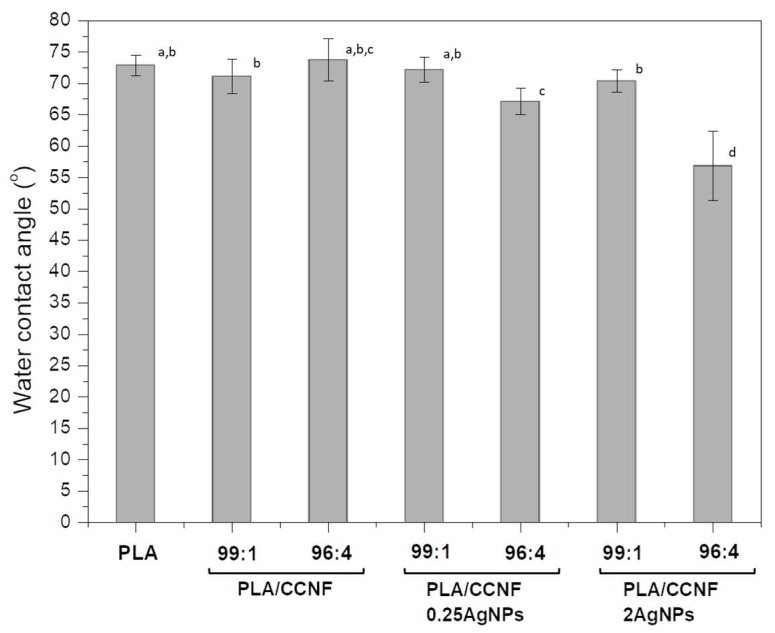
Water contact angles obtained for neat PLA and PLA reinforced with CCNF, CCNF/0.25AgNPs, and CCNF/2AgNPs films. Different letters (a–d) indicate significant differences (*p* < 0.05).

Water contact angles were measured for films and are presented in Figure 4. The water contact angle for neat PLA film was 72.9°, and it was lower than those obtained by Arrieta et al. (2015) [8]. The addition of CCNF or CCNF modified with AgNPs in relation to 99:1 didn’t influence the water contact angle in comparison with PLA film. The changes were visible only for films with higher amounts of nanofiller. The addition of pure CCNF in proportion 96:4 caused an increase in water contact angle and thus hydrophobicity. In contrast, the presence of CCNF/0.25AgNPs 96:4 and CCNF/2AgNPs 96:4 caused a significant decrease in water contact angle to 67.1° and 56.9°, respectively. This was the evidence of the increase in hydrophilicity of films containing larger amounts of silver nanoparticles. The hydrophobic/hydrophilic properties are important features of packaging materials, influencing their resistance to humidity. Previously, it has been shown that the addition of cellulose nanocrystals to PLA matrix causes a decrease of contact angle (from 80° to 70°) [8]. The values of water contact angle above 65° are considered as acceptable [8].

#### 3.2.5. Gas Permeability

Gas permeability is a critical issue regarding packaging composites. In some applications, where the reduction of oxidative processes is vital, good barrier properties to oxygen are important [4]. On the other hand, in the case of fruits and vegetable packages, a high transpiration rate of oxygen or carbon dioxide is a great advantage [26]. Here, the addition of CCNF to the PLA matrix increased the transpiration rate for all three tested gases reaching their highest value (Table 3). While for the composites of PLA with CCNF modified with AgNPs, the transpiration rates were lower but still larger than for neat PLA film. The best barrier properties, the closest to the values obtained for PLA, were obtained for PLA/CCNF/2AgNPs 96:4 composite but still were approx. 25% higher. Previously, rather the opposite effect was shown. Fortunati et al. (2013) showed that the addition of cellulose nanocrystals and silver nanoparticles decreased oxygen transmission rate even by 46%–60%; however, the best barrier properties had been achieved by composites where cellulose nanocrystals were modified by surfactant addition [10]. On the other hand, composites of PLA with cellulose microfibrils and silver nanoparticles have shown only little improvement in the barrier properties [4].

#### 3.2.6. Antibacterial Properties

The antibacterial properties of PLA/CCNF/AgNPs are presented in Table 4. The antibacterial properties were observed for all composite with CCNF or CCNF/AgNPs for *E. coli* and *B. cereus* but only in direct contact with composite. For neat PLA film, it was visible that bacteria were growing on the film surface, and the PLA was becoming opaque for each analyzed bacterial strain. For *Staphylococcus aureus*, there was practically no effect of all the analyzed nanocomposites; that is, it presented no growth only in the area of contact with the disc of the analyzed nanocomposites. Previous investigation of antibacterial activity of PLA reinforced with AgNPs has shown the inhibition effect and reduction of bacterial colonies, and that effect increases with increasing content of silver nanoparticles into composite [16]. Typically, the silver nanoparticles account for 0.5%–3% of composite weight [12,16,17]; however, Shameli et al. (2010) tested PLA films with the addition of AgNPs in the amount of 32 wt.% [27]. In the present study, the AgNPs were synthesized from 0.25 and 2 mM of AgNO_3_, resulting in the low content of silver nanoparticles. AgNPs were also bonded to nanocellulose surface, which, in turn, prevented their release and migration, resulting in antibacterial properties only in direct contact with the composite surface. This aspect had a positive result as the nanocomposite could not release AgNPs in contact with food (minimum value declared by EFSA is 0.05 of Ag/kg food). There was indirect proof that the bacteria didn’t grow on the surface, which was in contact with nanocomposite discs.

#### 3.2.7. Mechanical Properties

The mechanical properties reported in Table 5 were evaluated by tensile test. The addition of silver nanoparticles alongside cellulose nanofibers extracted from carrot showed a complex influence/effect on the mechanical properties of produced composites. Regardless of the addition of AgNPs, an increase in percentage shares of cellulose nanofibers in PLA matrix from 1% to 4% resulted in a decrease (significant or not) in Young’s modulus of composites. The observed effect was partially supported by other studies, showing the negative influence of an increase in the amount of cellulose fibers on Young’s modulus of the composite material [8,28]. However, other researchers have also reported results, which contradict current data, showing positive [17,29] or no significant influence [30] of fiber amount on the values of Young’s modulus of PLA composites. This indicates that the percentage shares of fibers in PLA composite may not be a simple scaling factor for values of Young’s modulus. For instance, Dong et al. (2017) showed that post extrusion annealing treatment led to 28 and 63% increase in tensile modulus and strength of the filaments, respectively [30]. Mokhena et al. (2018) reported that surface functionalization and processing methods (solution casting, extrusion, melt spinning) applied to PLA/CNF composites significantly alternated their mechanical properties, even in case of the same PLA-cellulose composition. Previously, in the case of ternary composites, the addition of cellulose nanocrystals (5%) and AgNPs (1%) has caused a decrease of the tensile strength (from 43 MPa to 29 MPa) but increase in Young’s modulus (from 2400 MPa to 2700 MPa) compared with pure PLA film [17]. Similar to other studies, the strain at break of PLA/CCNF composites was lower when compared with neat PLA and further decreased with the addition of cellulose [31]. The lowering of elongation at break is typical for thermoplastic composites [17]. The exceptions were reported in the case of the PLA/CCNF/2AgNPs composites, which showed a similar trend along with the increase in cellulose amount, but, in both cases, composites had higher values of strain at break than neat PLA. The value of strain at break depends on reinforcement dispersity in the matrix and thus reflects the interaction between nanofiller and matrix polymer. Therefore, higher values of strain at break for PLA/CCNF/2AgNPs suggested that nanofiller was better dispersed and interacted with matrix compared to other tested composites. Decreasing trends followed by an increase in the amount of cellulose were also reported for yield strength, yield strain, and tensile stress at the break for PLA/CCNF and PLA/CCNF/0.25AgNPs. Surprisingly, these trends were reversed in the case of PLA/CCNF/2.0AgNPs, which showed an increase in all three parameters alongside with the addition of the cellulose. The presence of AgNPs showed different types of beneficial effects on mechanical properties, depending on concentrations. Composites with 0.25 mM AgNPs showed the highest values for Young’s modulus, yield strength, and tensile stress at break among all tested materials. However, for composites with 2AgNPs, the average values for these parameters were lower compared to neat PLA. The higher amount of AgNPs also resulted in higher extensibility of produced materials. Composites with 2 mM AgNPs showed the highest values of strain at break compared to other composites and pure PLA. It could be concluded that relatively small addition of AgNPs resulted in an increase in stiffness and strengths of the material. However, further addition of silver up to 2 mM caused a decrease in both parameters with a simultaneous increase in the extensibility of the composite.

## 4. Conclusions

The nanocomposites based on PLA reinforced nanocellulose obtained from carrot pomace and silver nanoparticles were prepared by the solvent casting method. Previous papers presented ternary composites where silver nanoparticles and nanocellulose were used as separate additives to the PLA matrix. This was the first time when nanocellulose modified with silver nanoparticles was used as a reinforcing agent of PLA film. The obtained composites presented good transparency. The FTIR spectra showed that probably there was no interaction between matrix polymer and filler. SEM micrographs showed that the higher addition of filler (4 wt.%) improved the homogeneity of the composite. The most important functional features were studied, i.e., mechanical, hydrophilic, thermal, and antibacterial properties. DSC results showed that filler caused a decrease in degradation temperature, while the crystallization temperature of composites differed by 1–2 °C between composites. This might suggest that filler is well enough dispersed in the PLA matrix. The addition of CCNF modified with AgNPs caused an increase in hydrophilicity of composites in comparison with pure PLA or PLA/CCNF without AgNPs. The main factor increasing hydrophilicity was the amount of silver nanoparticles; therefore, the composites with PLA/CCNF ratio 99:1 were more hydrophobic. Moreover, nanocellulose itself caused higher gas permeability, and only for nanocomposites with CCNF and 2 mM AgNPs, the transmission rates of N_2_ and CO_2_ were on levels obtained for PLA film. While the oxygen transmission rates were higher, meaning that the addition of CCNF modified with silver nanoparticles made PLA film more permeable. All composites containing AgNPs exhibited antibacterial activity only for *Escherichia coli* and *Bacillus cereus* and only at the contact surface but did not show inhibition around films, which might indicate the lack of migration of nanoparticles. Finally, tensile tests showed that the addition of filler, especially CCNF modified with 2 mM AgNPs, resulted in increased flexibility of composite films. The composite tensile at break remained on the same level as for PLA film, but strains at break increased. This might suggest the best dispersion of filler in the PLA matrix.

## Figures and Tables

**Table 1:** Weight proportions of PLA, CCNF, and AgNPs in each composite. In this table, the initial concentration of AgNO_3_ in nanocellulose dispersion is given.

Acronym	PLA (g)	CCNF (g)	AgNO_3_ (mM)
PLA	0.500	-	-
PLA/CCNF 99:1	0.495	0.005	-
PLA/CCNF 96:4	0.480	0.020	-
PLA/CCNF/0.25AgNPs 99:1	0.495	0.005	0.25
PLA/CCNF/0.25AgNPs 96:4	0.480	0.020	0.25
PLA/CCNF/2AgNPs 99:1	0.495	0.005	2.00
PLA/CCNF/2AgNPs 96:4	0.480	0.020	2.00

PLA: Polylactic acid; CCNF: carrot cellulose nanofibrils; AgNPs: silver nanoparticles; AgNO_3_: silver nitrate.

**Table 2:** Thermal characteristics of neat PLA and PLA reinforced with CCNF, CCNF/0.25AgNPs, and CCNF/2AgNPs obtained from DSC curves.

Sample	*T*_g_ (°C)	*T*_c_ (°C)	*E*_c_ (J/g)	*T*_m_ (°C)	*E*_m_ (J/g)	*T*_d_ (°C)	*E*_d_ (J/g)
PLA	57.8	121.6	15.75	150.0	22.97	369.2	1123.90
PLA/CCNF 99:1	57.9	123.7	7.72	149.8	12.27	367.9	975.73
PLA/CCNF 96:4	58.6	122.3	7.49	149.7	9.98	362.1	1023.60
PLA/CCNF/0.25AgNPs 99:1	58.4	123.6	11.47	149.8	9.72	366.4	1002.90
PLA/CCNF/0.25AgNPs 96:4	58.8	123.5	8.48	149.7	14.44	354.9	823.03
PLA/CCNF/2AgNPs 99:1	57.8	124.9	7.2	149.2	9.78	363.4	865.58
PLA/CCNF/2AgNPs 96:4	58.0	124.4	9.99	148.6	11.34	348.4	668.55

*T*_g_: glass transition temperature, *T*_m_: melting temperature, *E*_m_: melting enthalpy, *T*_c_: crystallization temperature, *E*_c_: crystallization enthalpy, *T*_d_: decomposition temperature, *E*_d_: decomposition enthalpy.

**Table 3:** Transmission rates of tested gases: oxygen, nitrogen, and carbon dioxide. Different superscript letters (a,b,c,d,e) in each column indicate significant differences (*p* < 0.05).

Sample	Oxygen (mL·mm·min^−1^·m^−2^)	Nitrogen (mL·mm·min^−1^·m^−2^)	Carbon Dioxide (mL·mm·min^−1^·m^−2^)
PLA	39.24 ± 5.47 ^a^	155.91 ± 5.59 ^a,c^	0.30 ± 0.01 ^a^
PLA/CCNF 99:1	85.48 ± 2.93 ^a^	320.46 ± 13.44 ^a,b^	0.76 ± 0.39 ^a^
PLA/CCNF 96:4	63.00 ± 2.11 ^a,b^	235.65 ± 7.50 ^a,b,c^	0.55 ± 0.20 ^b^
PLA/CCNF/0.25AgNPs 99:1	50.58 ± 4.09 ^a^	189.55 ± 11.11 ^b^	0.64 ± 0.13 ^c^
PLA/CCNF/0.25AgNPs 96:4	66.58 ± 3.44 ^a,c^	246.81 ± 9.47 ^a,b,c^	0.80 ± 0.15 ^a^
PLA/CCNF/2AgNPs 99:1	47.95 ± 1.36 ^a,d^	182.07 ± 4.49 ^c^	0.31 ± 0.01 ^a,b,c,d^
PLA/CCNF/2AgNPs 96:4	49.00 ± 3.25 ^a,e^	183.16 ± 12.98 ^a,b,c^	0.34 ± 0.03 ^d^

**Table 4:** Antimicrobial activity of films obtained for neat PLA and PLA reinforced with CCNF, CCNF/0.25AgNPs, and CCNF/2AgNPs.

PLA:CCNF	*Bacillus cerreus*	*Staphylococcus aureus*	*Escherichia coli*
PLA	-	-	-
PLA/CCNF 99:1	-/+	-/+	-/+
PLA/CCNF 96:4	-/+	-/+	-/+
PLA/CCNF/0.25AgNPs 99:1	+	+	+
PLA/CCNF/0.25AgNPs 96:4	+	-/+	+
PLA/CCNF/2AgNPs 99:1	+	-	+
PLA/CCNF/2AgNPs 96:4	+	-	+

(+) an antibacterial effect at the contact surface with the composite, (-) lack of effect, (-/+) partial effect.

**Table 5:** Tensile properties of films obtained for neat PLA and PLA reinforced with CCNF, CCNF/0.25AgNPs, and CCNF/2AgNPs. Different superscript letters (a,b,c,d) in each column indicate significant differences (*p* < 0.05).

Sample	Young’s Modulus	Yield Strength	Yield Strain	Tensile Stress at Break	Strain at Break
	(MPa)	(MPa)	(%)	(MPa)	(%)
PLA	1940.9 ± 271.3 ^a^	21.6 ± 4.7 ^a,b,c,d^	1.92 ± 0.48 ^b,c^	26.39 ± 5.30 ^a,b^	2.76 ± 0.88 ^a^
PLA/CCNF 99:1	1923.7 ± 238.1 ^a^	19.9 ± 3.9 ^a,b,c^	1.31 ± 0.21 ^a^	28.66 ± 4.63 ^a,b,c^	2.19 ± 0.32 ^a^
PLA/CCNF 96:4	1769.4 ± 170.3 ^a,b^	16.9 ± 2.9 ^a^	1.20 ± 0.08 ^a^	23.45 ± 3.88 ^a^	2.06 ± 0.40 ^a^
PLA/CCNF/0.25AgNPs 99:1	2295.4 ± 203.1 ^c^	25.4 ± 6.3 ^d^	1.46 ± 0.40 ^a^	34.30 ± 7.16 ^c^	2.16 ± 0.48 ^a^
PLA/CCNF/0.25AgNPs 96:4	2199.4 ± 107.6 ^c^	23.8 ± 3.5 ^c,d^	1.37 ± 0.27 ^a^	31.36 ± 4.44 ^b,c^	2.06 ± 0.39 ^a^
PLA/CCNF/2AgNPs 99:1	1789.4 ± 253.5 ^a,b^	18.7 ± 1.9 ^a,b^	1.61 ± 0.36 ^a,b^	25.30 ± 2.81 ^a^	5.16 ± 2.33 ^b^
PLA/CCNF/2AgNPs 96:4	1659.8 ± 166.1 ^b^	21.7 ± 2.0 ^b,c,d^	2.04 ± 0.42 ^c^	26.57 ± 1.82 ^a,b^	3.13 ± 0.91 ^a^

## Supplementary Materials

The following are available online at https://www.mdpi.com/2073-4360/12/4/812/s1, Figure S1: Photo of PLA and nanocellulose modified with silver nanoparticles composites. The ratio PLA:CCNF is marked on the pictures, Table S1: The pre-culturing conditions for tested bacterial strains.
