# Supplementary material for: Evaluation of Nanocomposite Made of Polylactic Acid and Nanocellulose from Carrot Pomace Modified with Silver Nanoparticles
Source: Polymers (Basel). 2020 Apr 4;12(4):812. doi: 10.3390/polym12040812 (PMC7240409; doi:10.3390/polym12040812)
Supplement: Supplementary file 1 [file polymers-12-00812-s001.pdf]

Evaluation of Nanocomposite Made of Polylactic Acid and Nanocellulose from Carrot Pomace Modified with Silver Nanoparticles

Monika Szymańska-Chargot, Monika Chylińska, Piotr Pieczywek, Anna Walkiewicz, Giorgia Pertile, Magdalena Frąc, Krystian J. Cieślak and Artur Zdunek

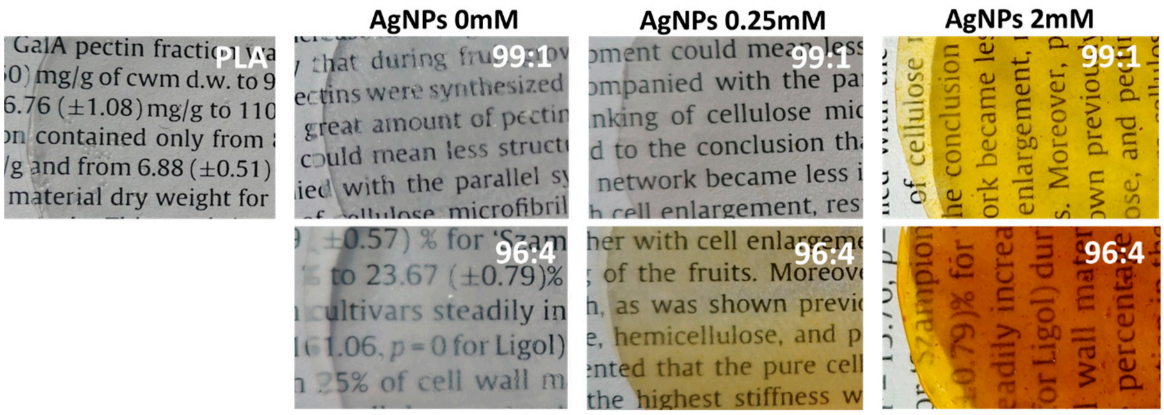

Figure S1. Photo of PLA and nanocellulose modified with silver nanoparticles composites. The ratio PLA:CCNF is marked on the pictures.

Table S1. The pre-culturing conditions for tested bacterial strains.

Microorganisms	Type/Family	Reference	Isolation Source	Accession Number	Medium	Temperature
Bacillus cereus	Gram-positive	LMEM B35/13	Waste digested	KJ531940 (NCBI)	Nutrient Broth/Agar	37 °C
Escherichia coli	Gram-negative	LMEM B4/164	Soil	MH176160 (NCBI)	Eijkman Broth/Agar	37 °C
Staphylococcus epidermidis	Gram-positive	LMEM B49/11	Organic waste	KJ020348 (NCBI)	Nutrient Broth/Agar	37 °C

LMEM - Laboratory of Molecular and Environmental Microbiology, Institute of Agrophysics, Polish Academy of Sciences.
